# Paradoxical effects of parathyroidectomy and sodium thiosulfate in a hemodialysis patient with calciphylaxis: a case report of persistent metastatic calcification

**DOI:** 10.3389/fmed.2025.1622599

**Published:** 2025-11-24

**Authors:** RuiLing Luo, Huiying Xu, Xuan Li, Zihao Zhu, Shuai Sun, Jinling Wang, XueXun Chen

**Affiliations:** Department of Nephrology, Affiliated Hospital of Shandong Second Medical University, Weifang, China

**Keywords:** end-stage kidney disease, sodium thiosulfate, calcific uremic arteriolopathy, tumoral calcinosis, multimodal therapy

## Abstract

Calcific uraemic arteriolopathy (CUA) is a rare and life-threatening condition characterized by vascular calcification, commonly observed in patients with end-stage kidney disease (ESKD). CUA is associated with a high 1-year mortality rate, ranging from 45 to 80%. Despite existing therapeutic options, managing CUA remains challenging with limited outcomes. This case report discusses a patient with ESKD undergoing long-term hemodialysis, who developed progressive CUA despite undergoing parathyroidectomy (PTX). Although sodium thiosulfate (STS) treatment promoted significant healing of skin ulcers, it had limited effects on tumoral calcinosis. This paradoxical clinical outcome highlights the complexity of managing CUA and underscores the need for a multimodal intervention strategy.

## Introduction

Calcific uraemic arteriolopathy (CUA) is a severe complication caused by medial arterial calcification and microvascular thrombosis, commonly seen in patients with end-stage kidney disease (ESKD) (1). The annual incidence of CUA in maintenance hemodialysis patients is ~3.49 per 1,000 patient-years (2). Major risk factors include obesity, diabetes mellitus, hyperphosphatemia, and long-term use of anticoagulants (3). The pathogenesis of CUA involves an interplay of mineral bone metabolism imbalance, medication exposures, and alterations in the vascular microenvironment (4).

Current management strategies for CUA primarily include sodium thiosulfate (STS), vitamin K supplementation, correction of mineral and bone metabolism abnormalities, and physical or interventional therapies. STS remains the cornerstone of therapy (3). Although STS has been shown to alleviate cutaneous manifestations, its efficacy in controlling visceral calcification is limited (5). These limitations may arise due to differences in response between active and mature calcification stages, the influence of other risk factors on disease progression, and the persistent pro-calcific uremic environment (6).

This case report presents a typical case of CUA progression, where, despite the control of secondary hyperparathyroidism, STS was effective in healing ulcers but ineffective against tumoral calcinosis. This case highlights the complex pathogenesis of CUA and the limitations of current treatment strategies.

## Case presentation

A 37-year-old male patient presented in March 2021 with widespread pruritus, left hip pain, and subcutaneous masses. Seven years ago, he sought medical attention due to nausea and vomiting, which led to a significant elevation in serum creatinine (1,319.0 μmol/L), resulting in a diagnosis of ESKD in the uremic phase, and initiation of thrice-weekly hemodialysis. One year prior to this visit, he developed generalized pruritus, left hip pain, and a mass, for which he self-administered sodium diclofenac gel. His medical history included renal anemia, renal osteodystrophy, secondary hyperparathyroidism, hypertension, herpes zoster, and chronic gastritis. His long-term medication regimen included Mecobalamin (150 mg, three times daily), Sodium Bicarbonate (1.0 g, three times daily), Calcium Carbonate (0.6 g, twice daily), Alfacalcidol (0.25 μg, nightly), Bailingtong (30 mg, every 8 h), Metoprolol (50 mg, twice daily), and Amlodipine (5 mg, twice daily).

**Figure 1:**
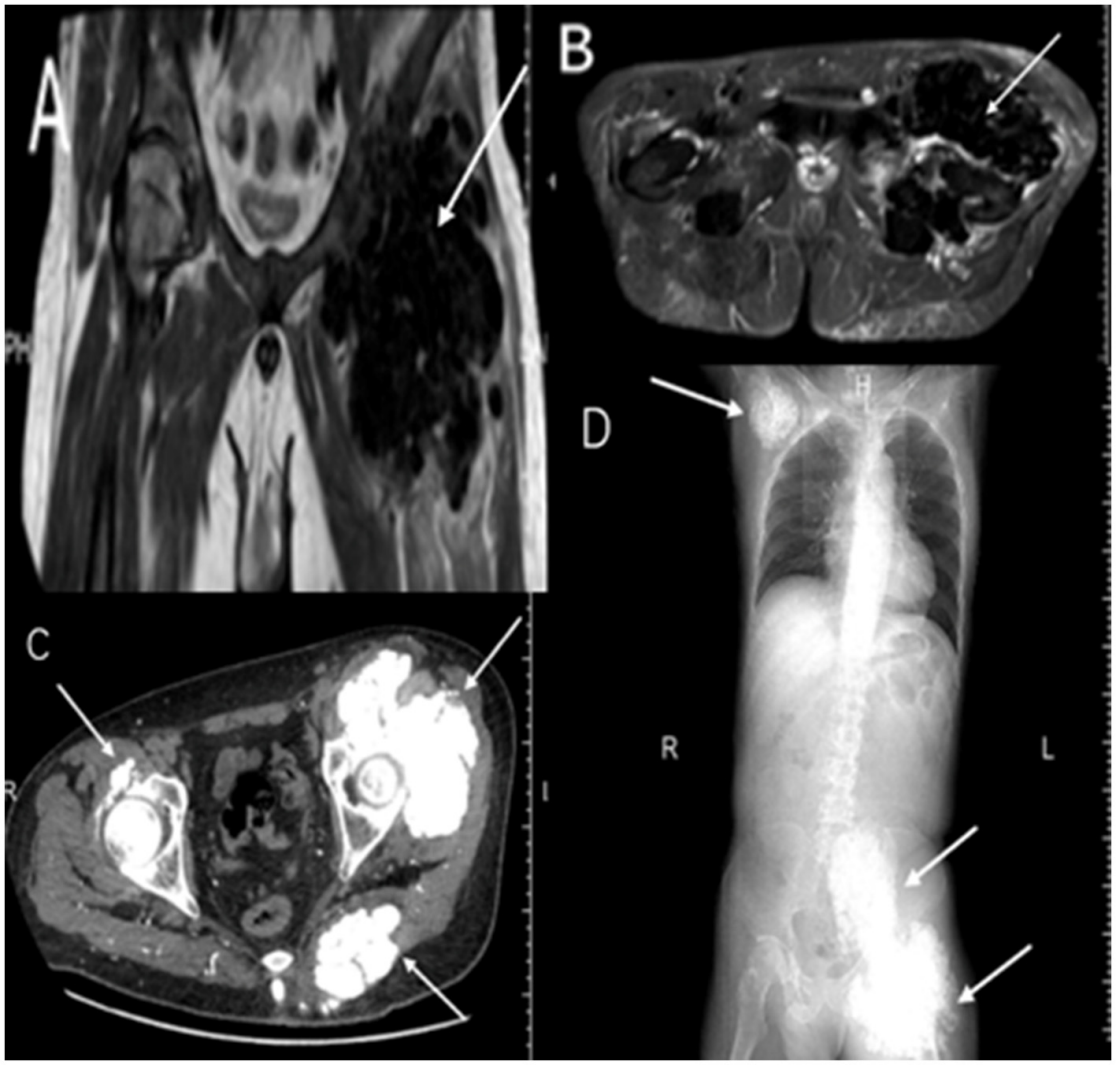
Serial imaging demonstrating calcification progression. **(A, B)** Pre-parathyroidectomy MRI shows sacrococcygeal and left femoral calcifications with perihippocampal edema (arrows). **(C, D)** Postoperative (19 months) CT reveals new spinal (C7-T1), thoracoabdominopelvic calcifications, and widespread tumoral calcinosis (shoulders, ribs, spine, femur, sacrum) with soft tissue calcification (arrows).

Upon admission, laboratory tests revealed elevated parathyroid hormone (PTH, 650.30 pg/ml) and serum phosphorus (2.85 mmol/L), indicating secondary hyperparathyroidism. Imaging showed metastatic calcifications in the sacral region and left proximal femur (Figures 1A, B), highly suggestive of calcific CUA. Given his medical history, the patient underwent total parathyroidectomy with autotransplantation. Postoperatively, iPTH decreased to 35.15 pg/ml, and serum calcium stabilized at 1.82 mmol/L, although hyperphosphatemia persisted (serum phosphorus 2.66 mmol/L).

After surgery, the patient continued thrice-weekly hemodialysis and was prescribed sevelamer for phosphorus control. However, due to persistent hypocalcemia and muscle cramps, he continued using calcium carbonate and alfacalcidol. Follow-up laboratory results showed that iPTH levels remained below 65 pg/ml, serum calcium fluctuated between 1.8–2.2 mmol/L, and serum phosphorus remained above 2.1 mmol/L, with CRP persistently >20 mg/L and albumin < 40 g/L. Other laboratory results are summarized in Table 1.

**Table 1:** Laboratory parameters at different treatment stages.

**Laboratory parameter**	**Pre-PTX**	**Post-PTX to pre-STS**	**Post-STS (1st course)**	**Post-STS (2nd course)**
Na mmol/	133.1	130–140	136.9	137.1
K mmol/L	6.78	5.3–6.5	5.25	5.74
Ca mmol/L	2.46	1.8–2.2	1.83	1.82
P mmol/L	2.85	>2.10	2.34	1.94
Ca × P (mg^2^/dl^2^)	86.94	>46.87	53.1	43.78
		(Est. up to ~68)		
PTH pg/ml	650.3	< 65	63.1	64.1
Cl mmol/L	88.2	90–95	90.2	97.7
WBC × 10^9^/L	9.38	< 10	10.09	8.42
Scr μmol/L	828	>800	655	585
BUN mmol/L	31.81	>26	26.86	25.54
Albumin g/L	40.8	< 40	36.2	32.1
FR-CRP mg/L	—	>20	63.63	76.89
PCT ng/ml	—	—	3.417	1.72

Reference ranges for laboratory parameters—sodium (Na) 137–147 mmol/L; potassium (K) 3.5–5.3 mmol/L; calcium (Ca) 2.04–2.71 mmol/L; phosphate (P) 0.85–1.51 mmol/L; chloride (Cl) 99–110 mmol/L; WBC 3.5–9.5 × 10^9^/L; FR-CRP (high-sensitivity C-reactive protein) 0–10 mg/L; albumin 40–55 g/L; procalcitonin (PCT) < 0.1 ng/ml. PTX, parathyroidectomy; STS, sodium thiosulfate).

**Figure 2:**
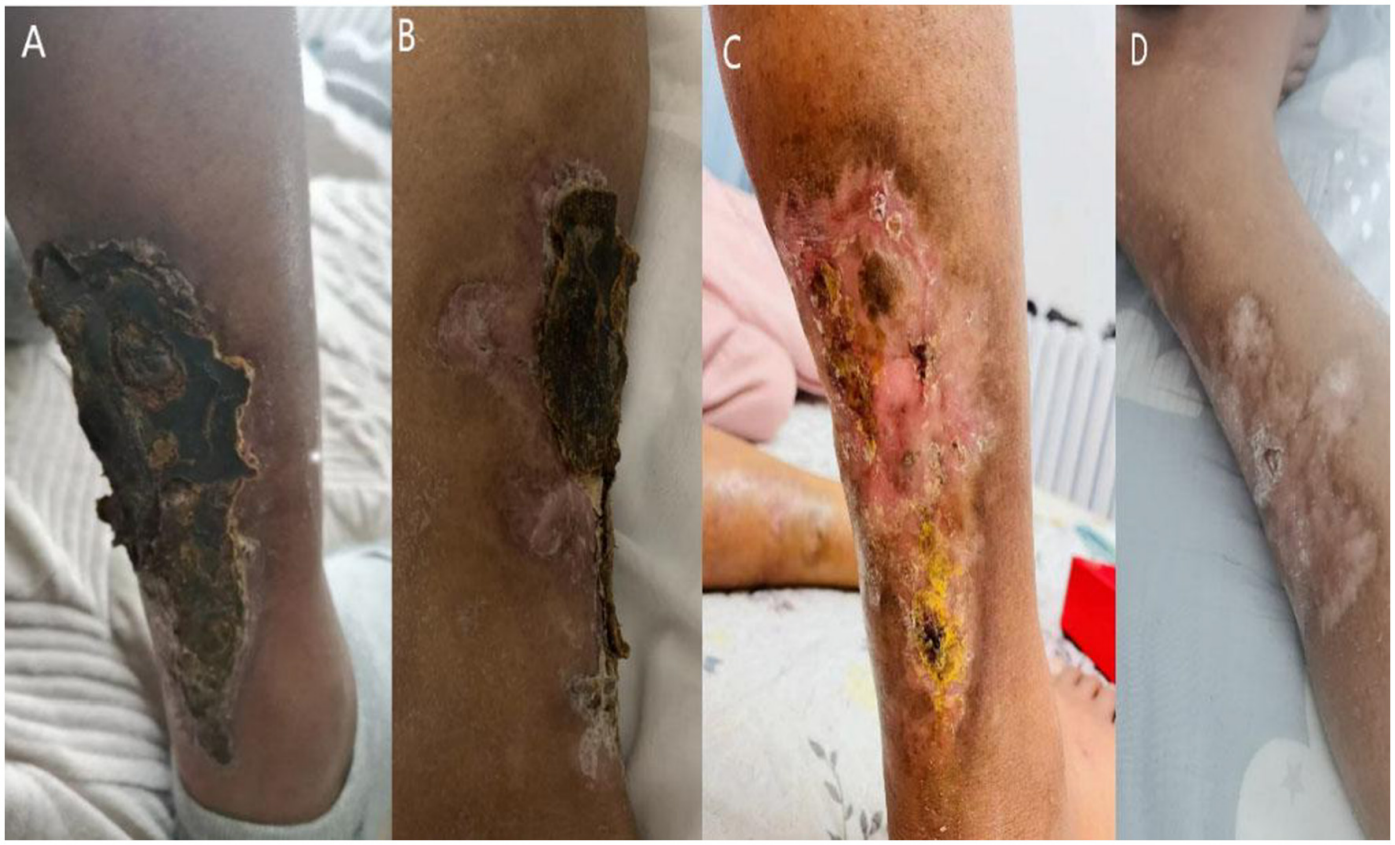
Cutaneous manifestations pre- and post-sodium thiosulfate (STS) therapy. **(A)** Pretreatment stellate ulcer (15 cm) with characteristic eschar at medial malleolus. **(B)** Significant wound reduction after initial STS course. **(C, D)** Complete epithelialization following second STS cycle.

Eighteen months after thyroid surgery, follow-up imaging revealed new extensive metastatic calcifications involving the shoulders, ribs, spine, femur, and sacrum (Figures 1C, D). In March 2023, the patient presented with worsening bilateral ankle ulcers, including a 15 cm stellate calciphylactic skin lesion (Figure 2A). Wound secretion cultures were negative, ruling out necrotizing soft tissue infection. Inflammatory markers were significantly elevated: white blood cell count 11.62 × 10^9^/L, high-sensitivity CRP 279.88 mg/L, PCT 14.88 ng/ml, and persistent hyperphosphatemia (serum phosphorus 2.19 mmol/L). Given the severity of his condition, the patient started intravenous STS therapy on March 29, 2023, following a stepwise dose-escalation protocol: starting at 3.5 g daily, increasing every other day to 5, 7, 8 g, and ultimately 10 g daily. The patient completed a 2-week course of treatment. In addition to STS, the patient received piperacillin-tazobactam for infection, Dan Shen injection for microcirculatory improvement, sevelamer for phosphorus control, esomeprazole for gastric protection, and optimized dialysis (using low-calcium dialysate). After the first STS course, CRP decreased by 77.3% (from 279.88 to 63.63 mg/L), and the ulcer area reduced by 42% (Figure 2B).

**Figure 3:**
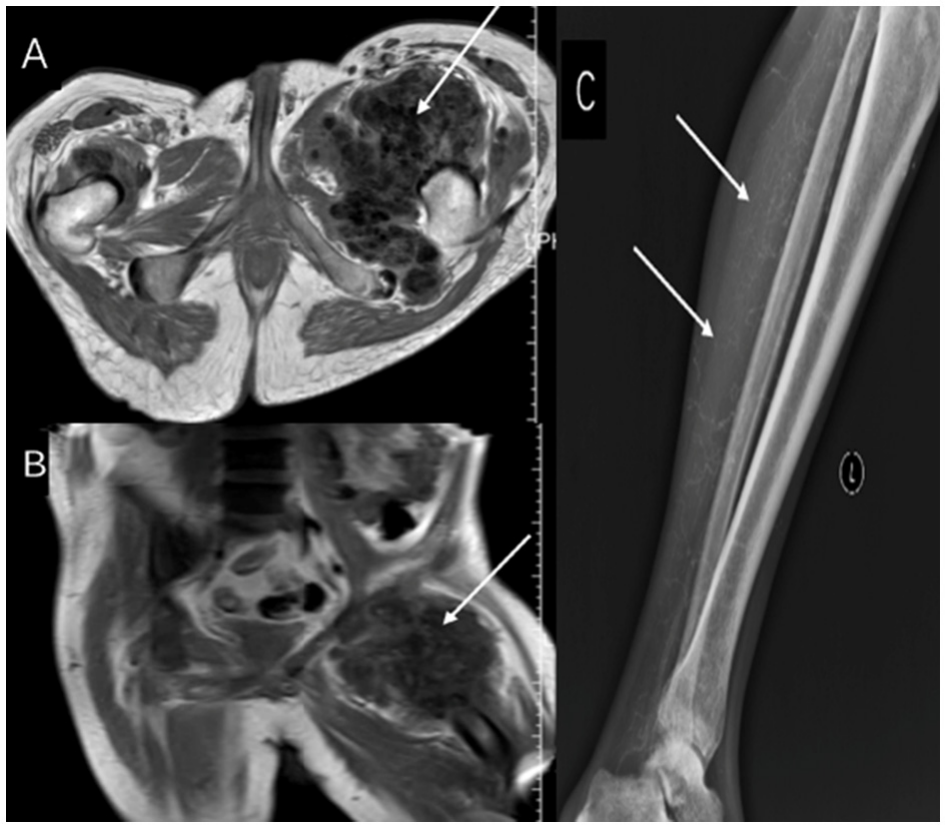
**(A, B)** MRI after STS therapy shows persistent sacrococcygeal/femoral calcifications (arrows), and **(C)** DR reveals new deposits in the knee region (arrow).

A second course of STS therapy was started on May 12, 2023, with a dose escalation from 5 g daily to 6, 8, and 10 g, maintained for 1 week, completing a total of 2 weeks. This resulted in complete epithelialization of the ulcer (Figures 2C, D), and no adverse events, such as gastrointestinal reactions, hypotension (BP 130–170/75–100 mmHg), acidosis, allergic reactions, or QTc prolongation, were observed. However, despite improvement in the skin lesions, imaging showed gradual calcification in the knee region, indicating the ongoing progression of deep tissue calcification (Figures 3A–C). The patient was subsequently transferred to another dialysis center, and follow-up by phone confirmed that the patient's lower extremity ulcers had not recurred and no new lesions had developed at other sites.

## Discussion

This case highlights a 37-year-old male with calcific uremic arteriolopathy (CUA) who experienced progressive metastatic calcification and severe skin ulceration despite standard interventions. Notably, the calcific lesions worsened even after parathyroidectomy (PTX), and extensive ulcerations developed. Treatment with intravenous sodium thiosulfate (STS) led to significant healing of the skin ulcers; however, serial imaging showed that the underlying “tumoral calcinosis” (large periarticular calcium deposits) continued to expand. This paradoxical outcome—ulcer healing without regression of calcified masses—underscores the complexity of CUA management and the need for multimodal therapy. It suggests that while STS can effectively promote wound healing, it has limited ability to dissolve mature calcium deposits, indicating that additional or alternative strategies are required to address the calcinosis component of CUA.

The diagnosis of CUA in this patient was based on characteristic imaging findings and clinical presentation. Given the extensive skin ulcers and the risk of infection, a skin biopsy was not performed. Magnetic resonance imaging (MRI) revealed widespread arterial calcification and subcutaneous calcium deposits. In light of the high clinical suspicion, the decision was made to continue calcification defense therapy. After reviewing the literature, we summarized eight published cases of CUA (see Table 2). These cases consistently emphasized that the diagnosis of CUA relies on a combination of clinical manifestations, medical history, risk factors, and imaging features, particularly when biopsy is not feasible (6).

**Table 2:** Summary of clinical cases and treatment outcomes in patients with CUA undergoing STS therapy.

**Study/case**	**Patient demographics**	**Underlying diseases**	**Diagnostic criteria**	**STS regimen**	**Adjunctive therapy**	**Clinical course and outcome**
Yalin et al. (32)	74 M	HTN, IGT, CVA, leg ulcer, long-term HD	CUA (bx, imaging)	25 g STS IV post-HD	Cinacalcet, wound care	Ulcers healed in 2 months, recurrence, died 3 years later
	58 M	DM, CAD, SHPT, long-term HD	CUA (X-ray, bx)	25 g STS IV post-HD	Cinacalcet 60 mg/day	Ulcers healed in 6 months
	70 F	CKD, renal transplant, steroids	Likely CUA (X-ray)	25 g STS IV (reduced dose)	Immunosuppressives, HD, nutrition	Ulcers healed in 3 months
Arrestier et al. (28)	49 F	SCD, heart disease, iron overload, ESRD	CUA (bx, imaging)	10 g STS IV, increased to 12.5 g	Cinacalcet, increased dialysis, nutrition	Skin lesions improved, died 1 year later
Burdorf et al. (29)	51 F	ESRD (HD), SHPT, IDDM, NYHA IV HF, AF, PAD	CUA (CT)	—	Cinacalcet, oxycodone, steroids	Improved, re-admitted for HF, transferred to long-term care
Bkiri et al. (30)	34 F	SLE, DM, hypercalcemia, SHPT, ESRD, CAPD	CUA (imaging, bx)	25 g STS IV, 3 × /weeks for 3 months	Increased dialysis, parathyroidectomy	Ulcers healed in 3 months, no change in dialysis modality
Lu (31)	40 M	HTN, SHPT, PD	Necrotic ulcers, vascular calcification	3.2–6.4 g/day STS IV, five courses	Lanthanum carbonate, celebrex	Ulcers healed after five courses, no new ulcers in 6 months
Albekery et al. (33)	42 F	DM, HTN, asthma, ESRD, PD	Right thigh ulcer, vascular calcification (X-ray)	25 g STS IV, 3 × /weeks for 2 weeks	Discontinued Ca/Vit D, low-Ca dialysis, pain medications	Ulcers healed in 6 months, pain relieved

HTN, hypertension; IGT, impaired glucose tolerance; CVA, cerebrovascular accident; CAD, coronary artery disease; SHPT, secondary hyperparathyroidism; DM, diabetes mellitus; CKD, chronic kidney disease; SLE, systemic lupus erythematosus; APS, antiphospholipid syndrome; SCD, sickle cell disease; ESRD, end-stage renal disease; CAPD, continuous ambulatory peritoneal dialysis; PD, peritoneal dialysis; CUA, calcific uremic arteriolopathy; STS, sodium thiosulfate; HD, hemodialysis; bx, biopsy; imaging, X-ray imaging; Ca, calcium; F, female; M, male; IDDM, insulin-dependent diabetes mellitus; NYHA IV HF, New York Heart Association class IV heart failure; AF, atrial fibrillation; PAD, peripheral arterial disease.

CUA occurring after PTX is relatively rare. After undergoing PTX, the patient's iPTH level decreased to below 65 pg/ml, and his serum calcium level remained between 1.8 and 2.2 mmol/L. Despite this, the patient still developed severe skin ulcers. This phenomenon suggests that the pathogenesis of CUA is highly complex, and a single factor cannot fully explain the progression of the disease (7, 8). These mechanisms include defects in calcification inhibitors such as carboxylated matrix Gla protein (c-MGP), fetuin-A, and inorganic pyrophosphate (PPi), leading to vascular microenvironment remodeling (9). Vascular calcification formation depends not only on calcium-phosphate imbalance but also on the actions of pro-calcification factors such as bone morphogenetic proteins 2 and 4 (BMP-2 and BMP-4) (10). PPi, an endogenous calcification inhibitor, can inhibit calcification by modulating the functions of ENPP1 (ectonucleotide pyrophosphatase/phosphodiesterase 1) and NT5E (CD73) genes (11–14). Mutations that lead to ENPP1 dysfunction result in hypophosphatasia, characterized by generalized arterial calcification of infancy (GACI), and plasma PPi levels are often reduced in patients with ESKD (11). Thus, even though calcium-phosphate product is a key culprit, the vascular micro-environment (with inadequate inhibitors) is primed for calcification in CUA.

Additionally, overexpression of BMP4 is closely associated with early vascular calcification in CKD patients (15). BMP-2 induces osteoblastic transdifferentiation of vascular smooth muscle cells (VSMCs) via the Wnt/β-catenin signaling pathway, promoting vascular calcification in CKD patients (16). In our patient, these osteogenic signals may have remained active or even been upregulated after PTX, continuing the calcific process independent of PTH levels.

We reviewed several case reports of CUA following PTX (17–21) (Table 3). Before surgery, the patient's PTH level exceeded 2,000 pg/ml, dropping to between 43 and 473 pg/ml after surgery. One hypothesis suggests that the rapid decline in PTH levels after PTX leads to a sharp decrease in bone metabolism, thus reducing calcium absorption. At this point, calcium cannot be effectively absorbed and stored in bones, resulting in excessive calcium-phosphate deposition in the vascular walls and soft tissues, ultimately leading to severe microvascular and subcutaneous extravascular calcification (22, 23). This phenomenon of calcium balance after PTX contributes to ectopic calcification (24). This also explains the potential reason for the expanded calcification defense range in this patient following PTX.

**Table 3:** Post-parathyroidectomy (PTX) clinical outcomes in calciphylaxis: case reports and treatment approaches.

**Study/case**	**Patient info**	**Underlying diseases**	**PTX indication**	**Pre-op PTH (pg/ml)**	**Post-op PTH (pg/ml)**	**Time to calciphylaxis**	**CUA diagnosis**	**Risk factors**	**Post-PTX treatment**
Wahab et al. (18)	33 M	ESRD, SHPT	SHPT	3,489	47	7 weeks	Skin biopsy	Unknown	Skin care, antibiotics, grafting
Karmegam et al. (17)	60 M	ESRD, AF, DM, HTN, gout	SHPT	4,191	184	4 weeks	Skin biopsy	Hyperphosphatemia, dialysis, heart disease, diabetes	Pain management, increased dialysis, STS, steroids
Sanha et al. (19)	26 F	ESRD, HTN, SHPT	CUA	>2,000	882 (intraop), 43 (da1), 118 (4 weeks)	3 weeks	Clinical	Female	STS, pain management, wound care
Dawson et al. (20)	50 M	ESRD, SHPT, Obesity	CUA	156	4.33	—	Skin biopsy	Obesity, Caucasian ethnicity, use of vitamin D analogs, HD	STS, surgical debridement, PTX, palliative care
Akad et al. (21)	38 M	ESRD, PKD, HTN, SHPT, HCV	SHPT	2,100	330–473 (varied)	Several months	CUA (MRI, clinical)	Hyperphosphatemia, hypocalcemia	Calcium-phosphate management, cinacalcet

PTX, parathyroidectomy; SHPT, secondary hyperparathyroidism; AF, atrial fibrillation; DM, diabetes mellitus; HTN, hypertension; PKD, polycystic kidney disease; HCV, hepatitis C virus; ESRD, end-stage renal disease; CUA, calcific uremic arteriolopathy.

It is noteworthy that, in addition to ESKD, the patient had multiple other risk factors that exacerbated calcification. These risk factors include hyperphosphatemia, hypoalbuminemia, obesity, and additional calcium and vitamin D supplementation. Extracellular inorganic phosphate (Pi) directly regulates VSMC-driven matrix mineralization (25, 26). The excessive accumulation of Pi is sufficient to induce VSMC senescence, further accelerating calcification (26). Moreover, Mozar's study indicated that high extracellular Pi concentrations inhibit osteoclast-like activity, leading to the transformation of calcium-phosphate deposits into more stable hydroxyapatite (HAP) crystals, which are surrounded by fibroblasts and compressed collagen fibers, making them difficult to absorb (27). This leads to persistent tumoral calcinosis (TC). Obesity and hypoalbuminemia are significantly associated with the occurrence of CUA (3). Common sites of calcification in uremic calciphylaxis are typically fat-rich soft tissues such as the abdomen, buttocks, and thighs (9). Obesity has been identified as a risk factor for CUA in multiple studies (3). A case-control study found that each 1 g/L reduction in serum albumin increased the risk of CUA by 1.33 times (3). Additionally, patients on long-term active vitamin D and its analogs are at higher risk of developing CUA (28). Among the cases we reviewed, three patients developed CUA post-PTX, and it is noteworthy that all of them received vitamin D analog treatment postoperatively due to hypocalcemia. This finding emphasizes the role of hypocalcemia correction as an exacerbating factor, as calcium supplementation may induce or worsen calcific lesions (19–21).

STS works through several mechanisms: first, its calcium chelation property forms highly soluble sodium thiosulfate-calcium complexes, significantly lowering the free calcium concentration in the vascular walls and surrounding soft tissues, thereby inhibiting the deposition of new calcium salts (29, 30). Second, its strong reductive properties effectively scavenge reactive oxygen species (ROS), preventing osteoblastic transdifferentiation processes induced by oxidative stress, thus slowing the progression of vascular calcification (31). Based on these mechanisms, STS therapy has shown significant effects in reducing all-cause mortality in CUA patients (29). However, in this case, although STS treatment led to rapid ulcer healing, MRI showed continued expansion of the calcification areas, highlighting the limitations of STS in reversing tumoral calcinosis. This is because STS primarily targets the active mineralization stage by inhibiting the nucleation of calcium-phosphate crystals. However, once mature hydroxyapatite deposits form stable calcific plaques with reduced surface energy and tightly cross-linked matrix proteins, STS becomes less effective in chelating and dissociating the calcification (4). Additionally, prolonged disease processes lead to the formation of hydroxyapatite crystals surrounded by fibroblasts and compressed collagen fibers, making them more difficult to absorb (32). This underscores the importance of early intervention in achieving therapeutic success.

Currently, there is no definitive consensus on the dosing regimen for STS in CUA treatment. Previous case reports have employed various administration protocols, with no consistent evidence proving that high doses or local STS administration are superior to low-dose intravenous administration in terms of efficacy. However, reports have indicated that high-dose intravenous STS is associated with an increased incidence of adverse drug reactions (1).

In addition to STS for CUA, other medications are also being used to target vascular calcification by alleviating underlying risk factors. Vitamin K1 (33), as a calcification inhibitor, suppresses vascular calcification by activating MGP, and studies suggest it holds potential in improving skin lesions and alleviating calcification in CUA patients. Magnesium-based drugs, such as magnesium citrate (34), inhibit HAP crystal formation by competing with calcium ions, thereby reducing the progression of vascular calcification. Furthermore, SNF472 (35), a novel calcification inhibitor, specifically binds to HAP crystals, preventing their deposition, and has shown promising clinical efficacy in trials, significantly reducing skin lesions in CUA patients. Cerium chloride (Fosrenol^®^) (36), a non-calcium-based phosphate binder, reduces intestinal absorption of dietary phosphate ions, indirectly inhibiting vascular calcification. In addition to these medications, optimizing dialysis regimens to control phosphate levels is also an effective strategy. The combined use of these approaches helps alleviate vascular calcification and related complications, improving patient outcomes.

## Conclusion

In conclusion, this case emphasizes several key lessons. First, CUA can occur even after elimination of hyperparathyroidism, due to the complex network of calcification drivers in ESRD patients. Second, early diagnosis (often clinical) and early intervention with therapies like STS are crucial to heal necrotic skin lesions and improve survival. Third, persistent calcific deposits may require additional novel therapies and risk factor modification beyond STS alone. And finally, a multidisciplinary, multimodal approach—including nephrologists, surgeons, dermatologists, nutritionists, and wound care specialists—is needed to tackle this life-threatening condition from all angles. By addressing the root causes (phosphate, PTH shifts, inhibitors deficiency) and utilizing emerging therapies (vitamin K, magnesium, SNF472, etc.), we can improve the outlook for patients suffering from calcific uremic arteriolopathy. The management of CUA remains challenging, but as this case illustrates, understanding its multifactorial nature guides us toward comprehensive care plans that can be life-saving for patients.

## Data Availability

The original contributions presented in the study are included in the article/supplementary material, further inquiries can be directed to the corresponding authors.
